# Improving Resistive Heating, Electrical and Thermal Properties of Graphene-Based Poly(Vinylidene Fluoride) Nanocomposites by Controlled 3D Printing

**DOI:** 10.3390/nano14221840

**Published:** 2024-11-17

**Authors:** Rumiana Kotsilkova, Vladimir Georgiev, Mariya Aleksandrova, Todor Batakliev, Evgeni Ivanov, Giovanni Spinelli, Rade Tomov, Tsvetozar Tsanev

**Affiliations:** 1 Institute of Mechanics—Bulgarian Academy of Sciences, 1113 Sofia, Bulgaria; kotsilkova@imbm.bas.bg (R.K.); vgeorgiev@imbm.bas.bg (V.G.); batakliev@imbm.bas.bg (T.B.); ivanov_evgeni@imbm.bas.bg (E.I.); gspinelli@imbm.bas.bg (G.S.); 2 Department of Microelectronics, Technical University of Sofia, 1756 Sofia, Bulgaria; rtomov@tu-sofia.bg (R.T.); zartsanev@tu-sofia.bg (T.T.); 3 Faculty of Transport Sciences and Technologies, University of Study “Giustino Fortunato”, 82100 Benevento, Italy

**Keywords:** GNP/PVDF nanocomposite, 3D printing, resistive heating, electrical performance, thermal properties

## Abstract

This study developed a novel 3D-printable poly(vinylidene fluoride) (PVDF)-based nanocomposite incorporating 6 wt% graphene nanoplatelets (GNPs) with programmable characteristics for resistive heating applications. The results highlighted the significant effect of a controlled printing direction (longitudinal, diagonal, and transverse) on the electrical, thermal, Joule heating, and thermo-resistive properties of the printed structures. The 6 wt% GNP/PVDF nanocomposite exhibited a high electrical conductivity of 112 S·m^−1^ when printed in a longitudinal direction, which decreased significantly in other directions. The Joule heating tests confirmed the material’s efficiency in resistive heating, with the maximum temperature reaching up to 65 °C under an applied low voltage of 2 V at a raster angle of printing of 0°, while the heating T_max_ decreased stepwise with 10 °C at the 45° and the 90° printing directions. The repeatability of the Joule heating performance was verified through multiple heating and cooling cycles, demonstrating consistent maximum temperatures across several tests. The effect of sample thickness, controlled by the number of printed layers, was investigated, and the results underscore the advantages of programmable 3D printing orientation in thin layers for enhanced thermal stability, tailored electrical conductivity, and efficient Joule heating capabilities of 6 wt% GNP/PVDF composites, positioning them as promising candidates for next-generation 3D-printed electronic devices and self-heating applications.

## 1. Introduction

In recent years, 3D printing by fused deposition modeling (FDM) has emerged as a leading manufacturing technology for a variety of industrial applications, in electronics [1,2,3], robotics [4,5], medical [6], and other fields, because it is cost-effective for prototyping and producing parts in small series. The success of 3D printing depends on fine-tuning materials to the needs of each application. A range of thermoplastic polymers with good mechanical properties and different textures are already commercially available for FDM application. However, there is a lack of commercial polymer composite filaments with specific physical properties and functionalities. Due to the growing interest in functional materials, conductive polymer nanocomposites have recently been studied for potential applications in FDM [7,8]. Graphene-based polymer composites have drawn significant interest for the fabrication of highly electrically and thermally conductive composite materials, due to the superior electrical conductivity (10^3^–10^5^ S·m^−1^) and thermal conductivity (~3000–4000 W·m^−1^ K^−1^) of pure graphene [9,10,11]. Most studies are focused on PLA-based composites incorporating graphene nanoplatelets and carbon nanotubes, suitable for 3D printing (FDM), for the design of structures with mechanical reinforcement, enhanced electrical and thermal conductivities, and electromagnetic shielding efficiency [12,13,14,15]. Despite the lab-scale efforts, however, there are only few commercially available functional filaments for 3D printing (FDM) [16,17].

PVDF is a thermoplastic material with excellent physical properties for piezoelectric and piezo-resistive applications in electronics and in the biomedical field. PVDF is a semi-crystalline polymer which shows electroactive properties due to its five different crystalline polymorphs: the non-polar α-form and the polar β, γ, δ, and ε-forms. The polymer molecules have a large dipole moment transverse to the polymer chain that determines its excellent electrical and piezoelectric properties [18,19,20,21]. With the inclusion of conductive nanoparticles, the piezo-resistive response of PVDF nanocomposites significantly increases, making these materials suitable for functional composite applications with large potential for force sensors, due to their mechanical and chemical resistance properties [22,23].

In general, the incorporation of multi-layer graphene nanoplatelets and graphite in polymers may lead to the formation of high-performance composites. However, property improvements are limited due to poor dispersion, the insufficient alignment of graphene nanoplatelets, and weak interactions at the graphene–matrix interface [24,25,26]. Therefore, graphene–polymer research is recently focused on the alignment of graphene to improve the electrical and thermal properties of the resulting composites because of the fact that the in-plane conductivity of a graphene sheet is much larger than its out-of-plane conductivity [24], but only limited successes have been achieved for graphene alignment in polymer matrixes due to the lack of efficient fabrication procedures [27]. 

Research works focusing on the thermo-resistive properties and Joule heating effect of polymer nanocomposites incorporating conductive nanofillers have attracted scientific interest due to their potential applications as heating elements, thermistors, smart materials, etc. [14,28,29,30]. However, when considering PVDF nanocomposites with carbon nanofillers, publications on the thermo-resistivity and self-heating effect of these materials are scarce. In our previous study [31], we investigated the effects of GNPs, MWCNTs, and their hybrid combinations on the crystalline structure and thermo-resistive performance of hot-pressed nanocomposites. The Joule heating temperature was successfully tuned by selecting the carbon nanofillers and the hybrid filler combinations. More research on PVDF-based nanocomposites is needed in order to understand the relationship between resistive heating and the electrical and thermal conductivity affected by carbon nanofillers.

Kausar et al. [32] reported that the 3D- and 4D-printed prototypes of polymer–graphene nanomaterials with programmable characteristics can be used for numerous applications; however, the choice of an appropriate printing material and the optimization of the printing parameters are indispensable. An important finding was that the integration of aligned graphene in 3D-printed objects generates continuous electron transfer pathways, leading to high electrical conductivity [33]. In our previous study [13], we found that the alignment of GNPs during the 3D printing (FDM) of polymer nanocomposites is due to the hydrodynamic slip of large GNP sheets in the nozzle flow. Several critical factors, such as layer height, infill density, and printing orientation, affect the mechanical properties and electrical conductivity of 3D-printed conductive polymer nanocomposites [34,35]. Tirado-Garcia et al. [36] developed PLA filaments highly filled with carbon black (CB) and found a dependence between printing direction and properties. However, studies on the advantages of 3D-printed polymer/graphene nanocomposites for resistive heating applications are scarcely reported [37]. So far, there is insufficient information on 3D-printable polyvinylidene fluoride–graphene nanocomposites [23]. Moreover, there is a lack of studies on the relationship between the printing parameters and multifunctional properties of printed structures. Future efforts are needed to relate the printing parameters, structural characteristics, and properties of PVDF/graphene nanocomposites for 3D printing applications.

In the present work, we explored the potential of 3D printing (FDM) and developed a novel printable 6 wt% GNP/PVDF nanocomposite with programmable characteristics for resistive heating applications. The key advantage of this study was the effect of the controlled printing direction (longitudinal, diagonal, and transverse) on the multifunctional properties, such as electrical and thermal conductivity, Joule heating, and thermo-resistivity, of the 3D-printed structures. The effect of the sample thickness, controlled by the number of printed layers, on the self-heating performance was investigated, offering insights into the optimization of properties. The results of this study will give evidence regarding the performance of the 6 wt% GNP/PVDF nanocomposite in 3D printing, self-heating, and thermal management applications.

## 2. Materials and Methods

### 2.1. Materials and Nanocomposite Preparation

Homopolymer grade poly(vinylidene fluoride) (PVDF) Kynar^®^ 721 (powder form) by Arkema (Philadelphia, PA, USA), with MFR 15 g/10 min (230 °C, 3.8 kg), a melting point of 168 °C, and a glass transition (*T_g_*) of −40 °C was used. Graphene nanoplatelets, with grade SE1233 (GNP), a purity ≥ 97%, a tap density < 0.1 g/cm^3^, an average diameter D50 ~35–50 μm, and SSA (BET) 400–600 m^2^/g were supplied by Sixth Element Material Technology Co. Ltd. (Changzhou, China).

The GNP/PVDF nanocomposite of 6 wt% filler content was prepared by the melt extrusion process. The filler content was selected above the electrical percolation threshold (EPT) in order to obtain a printable highly conductive nanocomposite. Our previous study determined the EPT of ~2 wt% [31]. The polymer and the filler were dried at 80 °C for 4 h in a vacuum oven; then, the PVDF powder was wrapped with the appropriate amount of GNP in a ball mill for 2 h at a speed of 70 rpm. Then, the wrapped powder was extruded in a twin-screw extruder Teach-Line ZK25T (COLLIN Lab & Pilot Solutions GmbH, Maitenbeth, Germany) at temperatures of 160–175 °C and a screw speed of 60 rpm. The developed nanocomposite filament was cut in pellets and further used for 3D printing of the test samples.

### 2.2. Controlled 3D Printing

The 3D printer used in this study was the Ender 5 Pro with a pellet extruder print head v4 MAHOR-XYZ (Mahor, Navarre, Spain) with a nozzle diameter of 0.8 mm, also known as fused granulate fabrication (FGF). The CAD models of test samples with length L = 30 mm and width W = 10 mm, with thickness of 2 mm and 0.8 mm, were created by the Fusion 360 software and exported as STL files (Standard Triangle Language, that is, the industry standard file type for 3D printing), then transferred to the Simplify3D software (Creative Tools AB, Halmstad, Sweden) to be printed. Optimal printing parameters were set—a print temperature of *T_p_* = 260 °C, a printing speed of *V_p_* = 1020 mm/min, and an infill parameter of 100%—to obtain the test samples. The PEI magnetic build platform was heated at *T_bp_* = 100 °C with a glue stick applied for better adhesion. The CAD models were printed layer-by-layer in a controlled 3D printing process. Three different printing directions were designed by controlling the raster angle of the printed layers: 0° for the longitudinal direction (3DP 0°); 45° for the diagonal direction (3DP 45°); and 90° for the transverse direction (3DP 90°) to the sample length. The raster size was 0.2 mm in height and 0.8 mm in width. The thickness of the samples was controlled by the number of printing layers. Thus, 10 printing layers were applied for the 2 mm sample thickness, while 4 printing layers were used for the 0.8 mm sample thickness.

### 2.3. Characterization Methods

Scanning electron microscopy (SEM) was performed to visualize the orientation of layers in the 3D-printed structures. Samples were cut longitudinally in liquid nitrogen and then gold-coated. SEM images of the cross-section were taken at 15 kV accelerating voltage and 110 mA emission current conditions with Tabletop SEM HIROX SH 4000 (Hirox Europe, Limonest, France) at different magnifications.

A transmission electron microscopy (TEM) analysis was performed by HR STEM JEOL JEM 2100 (Tokyo, Japan) with high-resolution operation with an acceleration voltage of 200 kV. The preparation of graphene samples was carried out by ultrasonic dispersion of GNP powder in ethanol. Then, a small quantity of the dispersion was placed on standard copper TEM meshes coated with an amorphous carbon membrane and dried at room temperature in a dust-free atmosphere. For the nanocomposite samples, thin sections were cut at room temperature with an ultra-microtome and placed on 400-mesh copper grids. The GNP sheets and the nanocomposite samples were examined at different magnifications to study the hierarchy of their structure from the micro- to nanoscale level.

Thermal analyses were performed to study the heat flow (DSC) and weight change (TGA) in the samples as a function of temperature under a controlled nitrogen atmosphere. A differential scanning calorimeter, DSC-Q20 (TA Instruments, New Castle, DE, USA), was used to monitor the heat effects associated with phase transitions of the polymer as a function of temperature in two heating runs, from 25 °C to 200 °C, at a heating rate of 10 °C/min, with subsequent cooling. From the DSC thermograms, the melting point (*T_m_*), melt crystallization temperature (*T_c_*), and endothermic melting enthalpy (Δ*H_m_*) were evaluated. The degree of crystallinity (%*χ_c_*) was calculated by Equation (1):

(1) $$\chi c\;\left(\%\right)=\frac{\Delta H_{m}}{\omega \cdot \Delta H_{mo}}*100$$

where Δ*H_m_* is the fusion/melting enthalpy (J/g), *ω* is the actual portion of polymer in the nanocomposite, and Δ*H_m_*_0_ = 104.7 J/g is the melting enthalpy for a 100% crystalline PVDF [32].

A thermo-gravimetric analysis was performed with TGA-Q50 (TA Instruments, USA) by heating from 25 to 800 °C with a ramp-up of 10 °C/min, under nitrogen flow. TG and DTG curves of weight loss and its first derivative were plotted versus temperature. Characteristic temperatures, such as the onset temperature at the start of weight loss (*T_onset_*) and the decomposition peak temperature (*T_peak_*), were evaluated. 

Electrical resistance (*R*) at a room temperature of 25 °C was tested by multimeter Keithley 6517B (Keithley Instruments, Cleveland, OH, USA) using the R-mode test. Before the tests, electrical contacts of silver paint, approximately 50 μm thick, were deposited on the upper and lower surface of the sample’s short ends, to ensure Ohmic contacts with the measuring electrodes. Commercial electrically conductive adhesive LOCTITE 3850 (produced by Henkel) with a volume resistivity of 0.00001 Ω·m was used for metallization. In the presence of metallization with silver, the contact resistance was considered negligible because it was much lower (of an order of mΩ) than the measured electrical resistance of the samples (of an order of Ω), as suggested and adopted in other studies in the literature [38]. The dimension of the test section ~20 × 10 × 2 mm^3^ was measured for each sample by a digital micrometer with an accuracy of 0.001 mm. Three samples were electrically characterized for each raster angle, and the average values were reported with a standard error of around ±10%.

The Joule heating (resistive heating) test was performed by an experimental setup consisting of a power supply (GW Instek, New Taipei City, Taiwan) providing the voltage (V) and measuring the current (I), while the heating temperature was controlled by a thermocouple type K connected to Keithley 6517B (Keithley Instruments, USA). Similarly to the upper test for the resistance measurements, electrical contacts of silver paint were realized on the short sides of the sample and connected to the power supply. The 3D-printed samples with the raster angles of 0°, 45°, and 90° were tested, with the test section of dimension ~20 × 10 mm^2^ and thicknesses of 0.8 and 2 mm, measured for each sample. Applying voltage to the sample, the local temperature evolution over time was measured by the thermocouple positioned at the center of the sample surface, ensuring close physical proximity to the surface by attaching it with a thermally conductive but electrically insulating LiPOLY AT910 tape (Taoyuan City, Taiwan). The goal was to have thermal coupling for accurate temperature readings without affecting the electrical measurements. Precautions were taken to ensure that no part of the thermocouple came into direct electrical contact with the sample to avoid interference with the current flowing through the sample during heating measurements. Data for the applied voltage and measured temperature, current, and time during four heating and cooling cycles were collected and recorded by the LabView Community edition software. Three samples were tested for each raster angle, and the average values were reported. 

Thermo-resistive property characterization was performed, as the resistance was measured by a multimeter Keithley 6517B (Keithley Instruments, USA), while the sample was heated up on a Peltier heating plate of the AR G2 rheometer (TA Instruments, USA) from 25 °C to 160 °C. The controllable temperature was set by the AR G2 (Trios software) with a step of 20 °C and recorded by the thermocouple type *K* to the Keithley 6517B instrument placed in the middle of the sample. Three samples were tested for each raster angle, and the average values were reported.

The thermal conductivity of the 3D-printed samples was tested with the Laser Flash Technique (LFA 467 Hyper flash, Neztsch, Hanau, Germany) in the temperature range of 25–110 °C, as limited from the heat deflection temperature of the PVDF (HDT ~110 °C at 264 psi, according to the TDS of the producer). Sample with a size of 10 mm × 10 mm and a thickness of 2 mm, printed with the three different raster angles, were tested with the heat flow perpendicular to the printing layers. The measurements were carried out in a standard sample holder (10 mm square) with three tests for each raster angle. The results report the average values. Prior to the measurements, the front and back sides of the samples were coated with graphite to enhance the emission/absorption properties of the samples. The specific heat was determined by the reference method. Therefore, the LFA was calibrated with a *C^p^*-standard (Pyroceram: Ø 10 mm, thickness 2 mm). The sample density at room temperature was measured using the buoyancy flotation method. From the resulting temperature excursion of the rear face measured with an infrared detector, thermal diffusivity and specific heat were both determined. Combining these thermophysical properties with the density value, the thermal conductivity was determined by Equation (2), as follows:

(2) $$\lambda \left(T\right)=\alpha \left(T\right)*C_{p}\left(T\right)*D(T)$$

where λ is the thermal conductivity [Wm^−1^ K^−1^], α is the thermal diffusivity [m^2^ s^−1^], Cp is the specific heat [JK^−1^⋅kg^−1^], and *D* is the bulk density [g·cm^−3^] of the composite. The Proteus^®^ Professional 8.6 software was used for data analysis and calculations of the thermal characteristics.

## 3. Results and Discussion

### 3.1. Nanocomposite Structure

**Figure 1:**
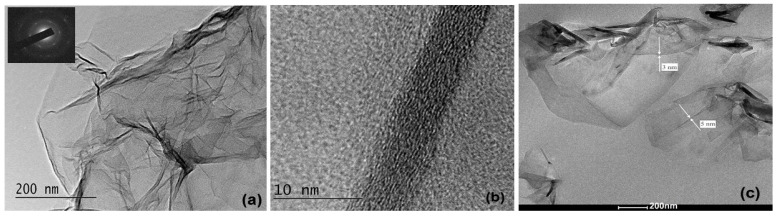
TEM images: (**a**) GNP filler surfaces with SEAD pattern (inset); (**b**) high-resolution TEM image of the GNP thickness showing the multi-layered structure of oriented graphene monolayers; and (**c**) exfoliated GNP nanostructures dispersed in the PVDF matrix. Arrows show the thickness of the exfoliated GNPs.

Figure 1 shows the TEM images of the commercial graphene nanoplatelets and the 6 wt% GNP/PVDF composite, confirming the nanometer thickness of the GNP sheets and their nanoscale dispersion in the PVDF polymer. Figure 1a shows the upper surface of a micron-size GNP sheet. The SAED pattern (inset) illustrates the multi-layer graphene structure in planes {100°} and {110°}. In Figure 1b, a high-resolution TEM image with higher magnification shows the GNP thickness of a nanoscale size < 10 nm, with a multi-layered structure of ~15–20 graphene monolayers having a relatively uniform orientation. In Figure 1c, the TEM image of the GNP/PVDF composite is presented, which shows partly exfoliated GNP sheets of 3–5 nm thickness with a diameter in the micron range, dispersed as nanostructures in the PVDF matrix. Large plate dimensions, a nanoscale thickness, and a high aspect ratio of GNPs are desirable for attaining a low electrical percolation threshold in nanocomposites. Our results confirm the suggestion of Müller et al. [39] that, during the extrusion processing of the GNP/PVDF composite, a polymer-assisted shear exfoliation of GNPs is achieved, but their lateral dimensions decrease due to breaking of the GNPs, compared to the initial particle sizes. Recently, the shear exfoliation of graphite nanoplates into nanostructures in the polymer was reported as a promising approach for developing multifunctional nanocomposites, but researchers agreed that a full exfoliation to individual graphene layers during the melt extrusion process could not be realized [39,40].

### 3.2. Thermal Analyses—DSC and TGA

**Figure 2:**
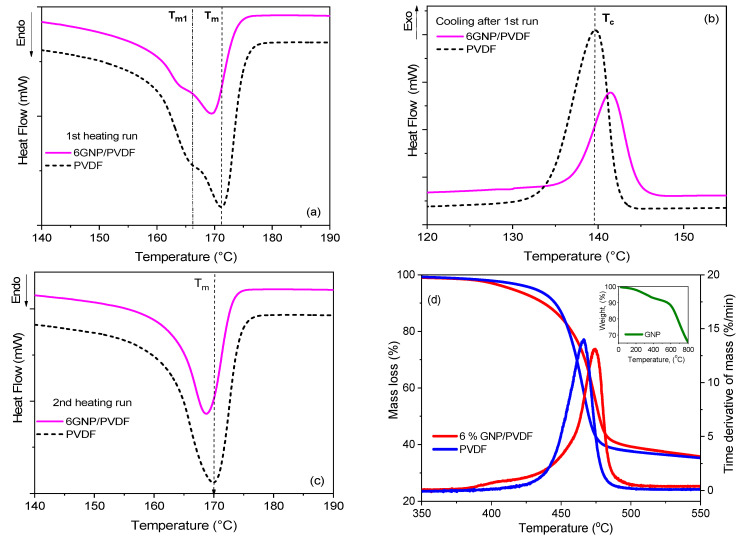
Thermal properties of PVDF and 6 wt% GNP/PVDF: DSC thermograms of heat flow vs. temperature at a scan rate of 10 °C/min, showing the first heating run (**a**), cooling cycle (**b**), and second heating run (**c**). The dash lines point the thermal transitions of the neat PVDF. In (**d**), the TGA/DTG thermograms of mass loss vs. temperature for the polymer and the nanocomposite are plotted, while the GNP thermogram is presented in the inset figure.

The DSC thermograms of the 3D-printed neat PVDF and 6 wt% GNP/PVDF nanocomposite are presented in Figure 2, and the thermal transitions are summarized in Table 1. The thermograms from the first heating run in Figure 2a demonstrate a double melting peak, which reveals the crystal structure formed during the 3D printing. The main melting peak *T_m_* appears at 171 °C for the neat PVDF and 169 °C for the GNP/PVDF nanocomposite, while a lower temperature shoulder, *T_m_*_1_ at 167 for PVDF and 163 °C for the composite, can be observed next to the main peak. In general, the graphene-based nanocomposite showed slightly lower melting temperatures compared to the neat PVDF. The cooling cycle after the first heating run in Figure 2b demonstrates the melt crystallization peak that appears at a higher temperature for the GNP/PVDF, but the peak intensity is smaller compared to that of the neat PVDF. This confirms the nucleation effect of the GNPs on the crystallization process of PVDF. Considering the second heating run in Figure 2c, there is only one sharp endotherm with a peak around 169–170 °C in the DSC curves for the second run of the nanocomposite and neat PVDF, respectively. This is usually associated with the melting of larger crystallites with a higher level of perfection which are formed from a melt crystallization during the cooling cycle [41,42]. The narrow melting peaks of the neat PVDF and the 6 wt% GNP/PVDF, found in the second heating run curves, reveal the homogeneity of the crystal structure formed during cooling with a constant rate of 10 °C/min.

A double endotherm of melting can be observed in the DSC curves of some semi-crystalline polymers [41], and there are two main interpretations of this phenomenon. One explanation is that the double endotherm is due to the melting of two different crystalline phases coexisting initially [42]. The other is that the lower-temperature endotherm does not correspond to the complete melting of a crystalline phase but rather to the melting of the imperfect crystalline region [43]. In our previous study [31], using XRD analysis, it was verified that the hot-pressed samples of the same PVDF homopolymer had a prevalent existence of the α-phase (~63%), while the β-phase content was very low, at ~0.2%. A slight increase in the β-phase fraction to ~3.1% was observed in the GNP/PVDF nanocomposites. Based on those findings, it might be assumed that the double endotherm observed in the first heating run of PVDF was mostly due to the α-phase crystalline structure with different crystal perfections formed during the 3D printing process, rather than a melting of two different crystalline phases. Meanwhile, a slight effect of melting of the coexisting small amount of β-phase could also be counted for the GNP/PVDF composite.

It is evident from Table 1 that the heat of fusion (∆*H_m_*) and the total crystallinity (*x_C_*) are decreased for the GNP/PVDF nanocomposite compared to the neat PVDF, for both data from the first and second heating runs. This effect can be explained by the large size and large surface area of the GNP sheets, which can restrict most of the polymer chains at a 6 wt% filler content, as the chains cannot move and orient to gain order and crystallization due to the presence of GNP. This finding confirms the results reported by Islam et al. [44] for PVDF/GO composites, according to whom, due to the constraining effect of GO on polymer chains, crystallinity decreases. The percentage of crystallinity is an important characteristic explaining the difficulties in 3D printing PVDF polymer and composites.

The TGA/DTG thermograms of the 3D-printed 6 wt% GNP/PVDF nanocomposite are presented in Figure 2d and compared with the thermograms of the neat PVDF and the GNP powder (in the inset figure). Table 1 summarizes data for the start of the weight loss (*T_onset_*) and the DTG peak of degradation (*T_peak_*). The PVDF and nanocomposite are highly thermally stable materials, as the start of PVDF degradation can be observed around *T_onset_*~450 °C, while the degradation peak appears around 470 °C. The nanocomposite demonstrates a 5–8 °C higher thermal stability compared to the neat PVDF due to the presence of the GNP filler. Regarding the filler (the inset in Figure 2d), the GNP degradation process may be formally divided into three stages. In the first stage, up to ~180 °C, the sample loses around 1.5% of its initial weight, likely due to the removal of adsorbed water from the GNP surface. In the second stage, from 180 to 365 °C, the continued weight loss up to ~7% suggests further decomposition or desorption of functional groups or impurities associated with the GNP. In the third stage, at 365–800 °C, the degradation of GNP starts at a *T_onset_* of 605.7 °C, and a rapid weight loss with a peak at 694.3 °C occurs, which is likely due to the breaking of the graphene platelets themselves [39]. The exact nature of this decomposition process can vary depending on the specific structure and defects of the GNP, potentially involving partial or even complete carbonization.

The DSC and TGA results were used to optimize the printing parameters, such as print temperature, heating temperature of the build platform, and printing speed, in order to avoid polymer degradation and minimize the peeling and distortion of the printed pattern due to polymer crystallization.

### 3.3. Morphology and Electrical Conductivity of 3D-Printed Structures

**Figure 3:**
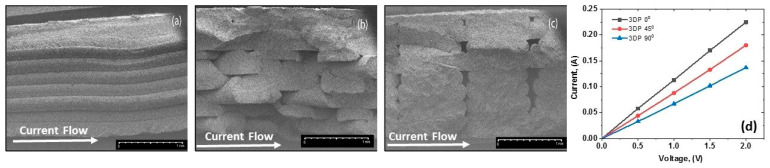
SEM micrographs of the cut surface of samples with different deposition directions: (**a**) longitudinal (3DP 0°); (**b**) diagonal (3DP 45°); (**c**) transverse (3DP 90°); and (**d**) voltage vs. current dependence, varying the printing directions. The magnification bar is 1 mm. The arrows show the current flow direction.

Figure 3a–c show the SEM micrographs of the surface of the longitudinally cut samples that are 3D-printed in three different directions with raster angles of 0° (3DP 0°), 45° (3DP 45°), and 90° (3DP 90°). The current flow direction is indicated with arrows in comparison to the direction of the printed layers. The 3DP 0° printed structure in Figure 3a presents horizontal layers of height ~0.2 mm stacked on top of each other in a continuous series. The orientation of the printed layers in this sample is in the same direction as the current flow. The bonding between layers appears to be consistent. In Figure 3b, the 3DP 45° samples are characterized by alternating regions of layers potentially revealing some voids or gaps where the layers intersect with the cut. The layers are ordered diagonally to the current flow. Figure 3c represents the 3DP 90° sample with lines printed perpendicularly to the sample length. At this position, layers of raster height ~0.2 mm and width ~0.8 mm are ordered perpendicularly to the current flow, and small voids between raster lines are noticeable for 100% infill printing. Figure 3d shows the current vs. voltage curves for the samples printed in the 3DP 0°, 3DP 45°, and 3DP 90° directions. The linear I–V dependence in the measurement range confirms the Ohmic coupling, which ensures that the current flows freely without additional potential barriers which would interfere with accurate measurements of the sample’s properties.

Electrical resistance, *R* [Ω], of the 3D-printed samples with a thickness of 2 mm was tested at a room temperature of ~25 °C, when comparing the three printing directions: longitudinal (3DP 0°); diagonal (3DP 45°); and transverse (3DP 90°). The resistivity (ρ) was calculated by the formula ρ = R × A/L [Ω·m], considering the resistance (R), the area of the cross-section (A), and the length of the test section (L) of the sample. The conductivity (σ) was calculated as the inverse of the resistivity: σ = 1/ρ [S·m^−1^]. Table 2 summarizes the average values of the resistance, resistivity. and electrical conductivity, with various printing directions. The highest electrical conductivity of 112 S·m^−1^ is observed for the samples with longitudinally printed layers (3DP 0°). In contrast, the conductivity is decreased progressively by increasing the raster angle of the 3D-printed layers, reaching values of 90.1 S·m^−1^ and 68.5 S·m^−1^ for 3DP 45° and 3DP 90°, respectively.

The orientation of the printing layers (0°, 45°, and 90°) has a significant impact on electrical conductivity due to how the material structure influences the current’s path. As the charge transport in the graphene–polymer composite above the percolation threshold depends mainly on particle contact and the tunneling effect, the alignment of large graphene nanosheets in the thin printing layers (0.2 mm height) enhances the surface contacts between GNPs, thus facilitating the current and heat flow:

- When the printed layers are oriented at 0°, relative to the direction of current flow (Figure 3a), the conductive pathways are continuous and aligned with the flow of electrons. This orientation minimizes resistance, as the current travels directly along the conductive pathways within the layers and there are fewer interruptions or boundaries that the electrons encounter, allowing a more efficient flow of current. Therefore, the 0° orientation results in the lowest resistivity in comparison to the 45° and 90° orientations (Table 2).
- At a 45° raster angle (Figure 3b), the conductive pathways are diagonal, meaning that electrons have to traverse boundaries between layers at an angle. This orientation introduces more interruptions and transitions between layers, increasing contact resistance and reducing slightly the overall conductivity compared to the 0° orientation. The current encounters more scattering and resistance due to the diagonal alignment, leading to an intermediate level of conductivity. This orientation could be useful in applications where a balance between conductivity and mechanical strength is desired, as it may offer slightly improved structural properties while still maintaining moderate conductivity.
- When the layers are oriented at 90° (Figure 3c), the current flow is perpendicular to the conductive pathways. This results in the lowest electrical conductivity because the current must cross multiple layer boundaries, each adding resistance and interrupting the flow of electrons. The 90° orientation introduces the highest amount of contact resistance as the electrons continuously jump across insulating or less-conductive interfaces. This is often unfavorable for applications requiring high conductivity but may have benefits in terms of mechanical reinforcement across layers.

### 3.4. Resistive Heating Performance

#### 3.4.1. Joule Heating Depending on the 3D Printing Direction

When an electrical current is applied to a semiconductor material, heat is generated, and this effect is known as Joule heating. The generated heat at the time at which the current is measured is calculated by Joule’s law, as per Equation (3):

(3) $$H=I^{2}R\;t$$

where *H* is the generated heat [J], *I* is the electrical current [A], *R* is the resistance [Ω], measured for the applied voltage *V* [V], and *t* is the time [s]. Based on Ohm’s law, *V* = *I* · *R*, the power (*P*) of the heating produced [W] is calculated by the common formula of $P=V\cdot I=I^{2}R$.

**Figure 4:**
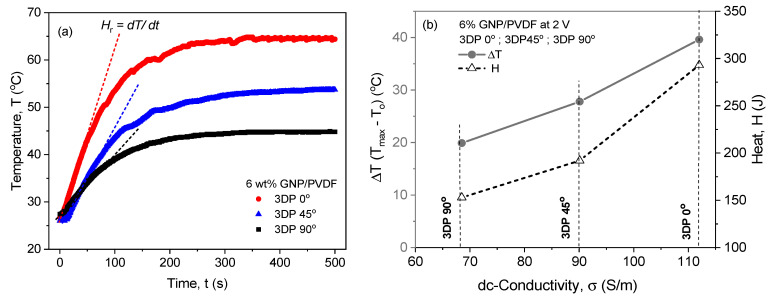
Comparison of (**a**) temperature vs. time and (**b**) temperature increase and heat vs. electrical conductivity of 6 wt% GNP/PVDF, varying the 3D printing directions—3DP 0°, 3DP 45°, and 3DP 90°—for 2 mm thick samples at an applied voltage of 2 V.

To generate internal Joule heating through nanofillers such as graphene, the fillers must be properly dispersed and stabilized to build percolated pathways. The 6 wt% GNP/PVDF material fulfills this requirement, showing stable and high dc-conductivity above the percolation threshold (Table 2). Figure 4 compares the Joule heating performance of 3D-printed nanocomposite samples of a constant thickness of 2 mm, varying the printing direction. In particular, Figure 4a shows the Joule heating temperature vs. time (0 to 500 s), comparing the longitudinal (3DP 0°), diagonal (3DP 45°), and transverse (3DP 90°) printing directions. All graphs highlight that the heating temperature increases steeply once the voltage (2 V) is applied, starting at 0 s, and, after ~100 s, the temperature growth rate gradually decreases and reaches the maximum equilibrium temperature (*T_max_*). The slope of the initial rapid temperature increase in the *T-t* curve is associated with the heating rate (*H_r_ = dT/dt*). The Joule heating *T-t* profiles of the longitudinal 3DP 0° printed samples demonstrate the highest *T_max_* of ~65 °C, but the values decrease for 3DP 45° (*T_max_*~54 °C) and 3DP 90° (*T_max_*~45 °C).

Table 2 summarizes the average values of the resistive heating characteristics for 2 mm thick samples, printed in the three directions, as follows: maximum temperature (*T_max_*), current (*I_max_*), power of heating (*P*), generated heat (*H*) at t = 500 s, heating rate (*H_r_ = dT/dt*), heat efficiency, *H_eff_* = (1 − (T_o_/T_max_)) × 100, in %, where *T_o_* = 25 °C is the initial temperature. As can be seen, the longitudinally printed sample (3DP 0°) demonstrated the highest heating properties, followed by the diagonally printed sample (3DP 45°), with lower values. Meanwhile, the lowest values of Joule heating were obtained for the transversally (3DP 90°) printed structure. As mentioned above, the orientation of printing layers (0°, 45°, and 90°) has a significant impact on conductivity due to how the material structure influences the current path and heat flow. The alignment of large graphene nanosheets in the thin printed layers enhanced the surface contacts between GNPs and thus facilitated both current and heat flow.

Figure 4b plots the temperature increase, Δ*T* (=T_max_ − T_o_), °C, and the generated heat (*H*), calculated by Equation 3, versus dc-conductivity (*σ*), at *T_o_* = 25 °C. It is evident that there is a relationship between the Δ*T*–*σ* and the *H*–*σ* functions for the 6 wt% GNP/PVDF, which depends strongly on the printing directions. The slope of both functions increases steeply from the 3DP 90° to the 3DP 45° conductivity values, while it becomes smoother for the conductivity of the 3DP 45° to 3DP 0° printed directions. The change in the slope observed at *σ_DC_*~67 S·m^−1^ impedes the hypothesis that 3DP 45° can be counted as the optimal parameter that triggers heating, together with the best one, 3DP 0°, when, for example, a good mechanical performance is required. The highest electrical conductivity and resistive heating properties obtained for 3DP 0° are probably due to the alignment and self-assembly of the large GNPs along with the direction of the current flow. In contrast, the transverse printing direction (3DP 90°) orients the GNP layers in a direction transverse to the current flow, which suppresses resistive heating. Based on these dependences, a nanocomposite with the appropriate 3D printing direction can be selected depending on the conductivity and the applied voltage in order to reach the required temperature increase. These relationships are weakly discussed in the reported literature, mainly in relation to percolation [43]. Therefore, our results can be useful in investigating the practical advantages of Joule-heated polymer nanocomposites for electronics and stimulus-responsive applications.

#### 3.4.2. Repeatability of Joule Heating

**Figure 5:**
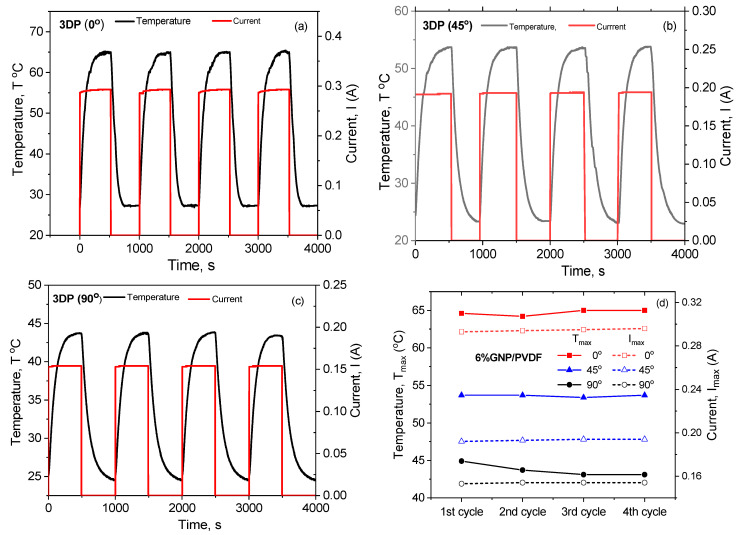
Temperature vs. time for four-cycle heating–cooling test of 6 wt% GNP/PVDF samples at an applied voltage of 2 V with various printing directions: (**a**) longitudinal 3DP 0°, (**b**) diagonal 3DP 45°, and (**c**) transverse 3DP 90°. (**d**) Repeatability of the maximal temperature and current in the four heating–cooling cycles for the three printing directions.

The kinetics of continuous heating and cooling were analyzed by a cycling test of four cycles, as shown in Figure 5. The heating and cooling cycles are compared for the 3D-printed 6% GNP/PVDF nanocomposite, varying the printing direction controlled by the raster angle—0° 3DP in Figure 5a, 45° 3DP in Figure 5b, and 90° 3DP in Figure 5c—for the 2 mm thick samples by applying voltage of 2 V. For all samples, the heating cycles are fixed to 500 s, and, consecutively, after switching off the electrical power supply, the temperature decreases to room temperature. The curves in Figure 5a–c can be divided into three sections: (i) temperature growth (heating 0–100 s), (ii) the region of equilibrium (maximum) temperature (100–500 s), and (iii) temperature decay (cooling to room temperature). Thus, under the same applied voltage of 2 V, the magnitudes of the maximum temperatures were nearly the same for all four cycles.

Figure 5d demonstrates the repeatability of the maximal heating temperature (*T_max_*) and current (*I_max_*) in the four-cycle test with various 3D printing directions. In general, the repeatability is very good for the parallel (3DP 0°) and diagonal (3DP 45°) printing directions, while a deviation of ~2 °C is observed in the maximum temperature between the first and second heating cycle, but, between the third and fourth cycle, the repeatability becomes stable. The plateau values of *T_max_* in the four-cycle repeatability test in Figure 5d allow us to predict that multiple Joule heating–cooling is possible if the *T_max_* is below the temperature of the end of the melt crystallization peak of the nanocomposite (<135 °C) in Figure 2b. This is evidently due to the absence of any thermal transitions of the polymer structure in the temperature region of 20–135 °C.

#### 3.4.3. Joule Heating Controlled by the Number of Printed Layers

**Figure 6:**
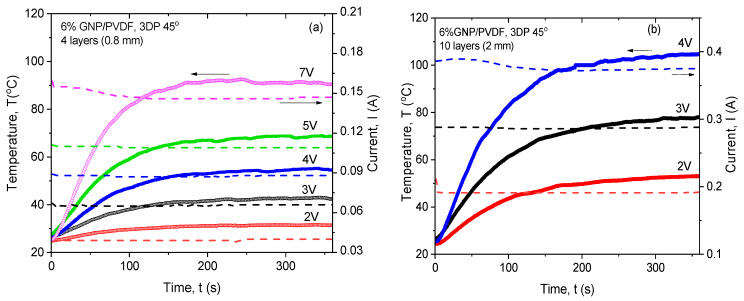
Temperature and current vs. time for 6 wt% GNP/PVDF, for the diagonally printed samples (3DP 45°) with (**a**) 4 printed layers (0.8 mm thick) and (**b**) 10 printed layers (2 mm thick), varying the applied voltage.

The Joule heating effect controlled by the number of printed layers was studied for the 3DP 45° samples. Indeed, the 45° printing direction is well known as the optimal orientation of the layers for 3D printing without support, so-called bridging, as well as for good mechanical reinforcement [34,36]. Therefore, the 3DP 45° printing direction was chosen here to verify the resistive heating behavior of the samples with better mechanical reinforcement. The temperature increases vs. time curve was measured for different applied voltages, when the number of the 3D printing layers controlled the sample thickness. In Figure 6a, the *T*−*t* and *I*−*t* curves of the 4 printed layers with a raster height of 0.2 mm (~0.8 mm thickness) are presented with various applied voltages, from 2 V to 7 V. Meanwhile, in Figure 6b, the 10 printed layers with a raster height of 0.2 mm (~2 mm thickness), with varying voltages from 2 V to 4 V, are compared, in order to keep the maximal heating temperature below the HDT (110 °C) of the PVDF. In general, the temperature and current at the equilibrium plateau (at *t*~300 s) increased with the increase in the applied voltage, with values which depended strongly on the number of printing layers. The thicker samples (2 mm) with 10 printed layers produced higher *T_max_* and *I_max_* at the same applied voltage, compared to the thinner samples (0.8 mm) with 4 printed layers. The results are summarized in Table 3.

**Figure 7:**
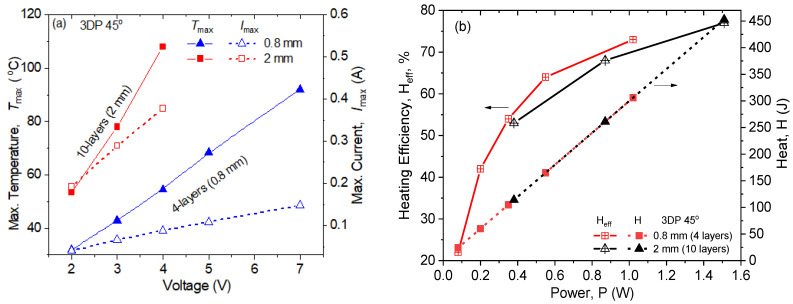
Comparison of (**a**) maximum heating temperature and current vs. applied voltage and (**b**) generated heat and heating efficiency vs. power for the 3DP45° samples of the 6 wt% GNP/PVDF nanocomposite, with a controlled number of printed layers (4 layers, 0.8 mm thick; and 10 layers, 2 mm thick).

Figure 7 compares the maximal values of the heating characteristics vs. applied voltage and power. In Figure 7a, the *T_max_*–*V* and *I_max_*–*V* curves are plotted. As can be seen, when the printing layers increase from 4 to 10, an enhancement in the *T_max_* and *I_max_* can be observed at the same applied voltage, and the slope of the curves is increased by increasing the number of layers. For example, the thinner sample with four layers (h = 0.8 mm) reached the equilibrium temperature *T_max_* = 92 °C at a voltage of 7 V, which produced a temperature increase of 67 °C with respect to the room temperature (Δ*T = T_max_* − 25 °C). Meanwhile, the thicker sample with 10 layers (*h* = 2 mm) reached *T_max_* = 109 °C at a voltage that was about twice as low as 4 V, with a Δ*T* = 83 °C, which was a considerable temperature increase at such a low voltage, considering data in the literature [31]. A linear relationship between *T_max_*–*V* and *I_max_*–*V* was observed for both numbers of printing layers, which allowed us to predict the temperature of self-heating and current by increasing the applied voltage.

In Figure 7b, the heating efficiency (*H_eff_*, %) and generated heat (*H* (J)) vs. power (*P* (*W*)) (at *t* = 300 s) are plotted. As can be seen, the heat (*H*) increased linearly by increasing the power, while the *H_eff_*–*P* dependence tended to plateau at the highest power values. Importantly, the generated heat was not influenced by the sample thickness at a fixed power, so it was one and the same for the 10-layer and 4-layer samples. If we consider the heat efficiency, however, the thinner samples (0.8 mm) seemed to demonstrate higher heat efficiency compared to the thicker ones (2 mm), at the same power. 

From an electronic perspective, an electrically controlled element characterized by a thin-film structure consisting of four layers presents a more advantageous and efficient configuration. This design enables the attainment of temperatures approaching ~100 °C with a current that is more than twice as small compared to equivalently functioning elements in a thick-film structure composed of ten layers. If the element is utilized in heating applications, it can be managed by electronic components with reduced power requirements, leading to a lower overall energy consumption. At an input electrical power of only 1 W, the thinner structures can generate a significant amount of heat (306 J) at t = 300 s, with a heating efficiency of 73%. Meanwhile, the thicker structures, at 1 W of input power, generate the same heat with a slightly lower heating efficiency of 70% (Figure 7b). The higher heating efficiency at low power consumption levels demonstrates the effectiveness of the four-layer thin-film design in optimizing thermal performance and energy efficiency in comparison to the ten-layer design.

### 3.5. Thermo-Resistivity and Thermal Properties

#### 3.5.1. Resistance–Temperature Analysis

**Figure 8:**
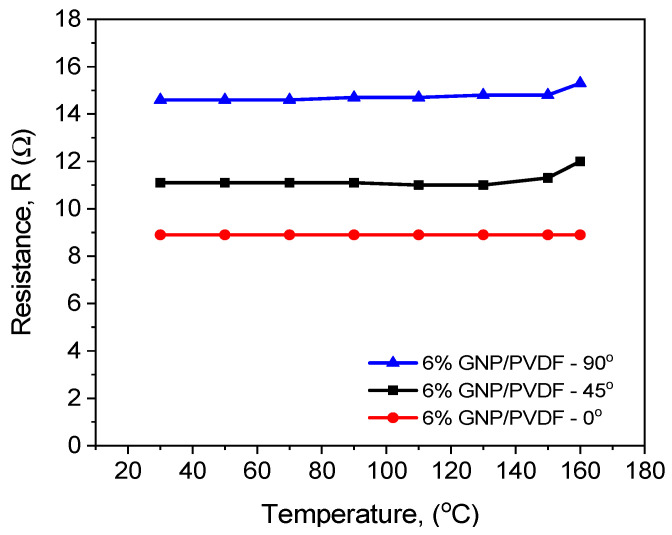
Resistance vs. temperature of the 6 wt% GNP/PVDF composites with various printing directions of 3DP 0°, 3DP 45°, and 3DP 90°.

In Figure 8, the measured electrical resistance as a function of temperature in the range of 25 to 160 °C is plotted for the 3D-printed 6 wt% GNP/PVDF composite, comparing the three printing directions. The average values of *R* are presented, with a standard deviation error of around 10%. In general, the resistance of the 3DP 0° sample demonstrates the lowest values, followed by 3DP 45° and 3DP 90°, with much higher resistance values, respectively. The resistance–temperature profiles show no change in resistance by increasing the temperature for the longitudinally printed sample (3DP 0°) in the whole temperature range. Therefore, the large GNP at 6 wt% content formed a denser network in the PVDF matrix, leading to low resistance and low sensitivity to temperature. However, the diagonally (3DP 45°) and transversely (3DP 90°) printed samples showed an electrical resistance increase with increasing temperature in the range of 150–160 °C. This slight PTC effect is attributed to the thermal expansion of the crystalline polymer during melting (see Figure 2a,c), which resulted in a local breakdown of the GNP conductive network. The mechanism of nanofiller rearrangement can be applied here, which states that changes occur in the gathering and/or orientation of nanofillers when the polymer matrix starts to melt [45].

#### 3.5.2. Heat Flow Analysis

**Figure 9:**
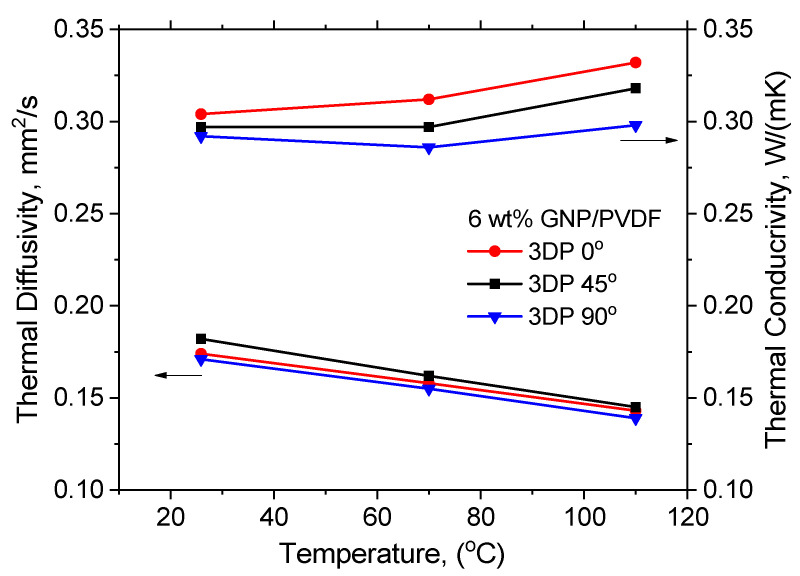
Thermal diffusivity and conductivity of the 3DP samples of 6 wt% GNP/PVDF vs. temperature, with various printing directions.

For self-regulating heating elements, it is essential to ensure that the heat generated is effectively conducted throughout the material to achieve uniform heating. It affects the overall performance and durability of the heating elements. Materials with higher thermal conductivity can dissipate heat more effectively, reducing the risk of thermal degradation and prolonging the lifespan of the heating element. Therefore, the thermal diffusivity of the 3D-printed samples was measured in the temperature range of 25–110 °C (the HDT) to evaluate the temperature perturbation transfer rate inside the 3D-printed samples. Thermal conductivity was determined to demonstrate the material’s intrinsic ability to transfer or conduct heat. Figure 9 compares the thermal diffusivity and thermal conductivity of the samples with differently oriented printing lines. The average diffusivity values with a small standard deviation below ±0.0005 mm^2^/s between individual shots at a given temperature are presented.

As seen in Figure 9, the thermal diffusivity decreased linearly by increasing the temperature for all samples, but the diffusion values did not differ significantly for the three printed directions. This is because the heat transfer was measured perpendicularly to the anisotropic and aligned GNP sheets. If we were to consider the thermal conductivity, it was initially almost independent from the temperature increase, but at higher temperatures above 70 °C, a small rise in thermal conductivity was observed. There was a slight difference in thermal conductivity with varying the printing direction; thus, the highest thermal conductivity in the whole temperature range was observed for 3DP 0°, followed by 3DP 45° with a slightly lower conductivity, and, finally, 3DP 90°, with the lowest conductivity values. The observed temperature dependence for thermal diffusivity and conductivity was due to increased molecular motion. As the temperature increased, the polymer chains within the composite acquired more kinetic energy, resulting in enhanced molecular movement, which disrupted the ordered structure of the polymer matrix impeding heat conduction through the material. Additionally, as the temperature increased, the difference in the coefficients of thermal expansion (CTE) between PVDF and GNP caused internal stresses in the composite. This can cause the GNP particles to detach from the matrix polymer [46], resulting in a decrease in the contact area between the filler and the matrix, which worsen the thermal conductivity. Due to the large differences in CTE (a negative CTE for GNP [46] and a positive for the polymer), micro-cracks can form at the polymer–graphene interfaces, making the interface less efficient in heat transfer. 

## 4. Conclusions

This work developed a novel 6 wt% GNP/PVDF composite by extrusion processing, characterized by high electrical conductivity, enhanced thermal stability, and strong and repeatable Joule heating efficiency, by applying a low voltage, which could be proposed as a multifunctional material for 3D printing (FDM) for self-heating, thermo-resistive, and high-temperature applications. In DSC and TGA analyses, the thermal properties were characterized and used to optimize the print temperature and the heating of the building platform in order to avoid polymer degradation and minimize the printing problems due to the fast crystallization of PVDF. We found that the orientation of the printing layers (0°, 45°, and 90°) had a significant impact on the electrical conductivity and Joule heating properties due to the influence of the layers’ structure on the current’s path and heat dissipation. The 0° printing orientation was in line with the current flow, and it provided the highest electrical conductivity and Joule heating efficiency, as the conductive pathways were continuous and aligned with the flow of electrons. The diagonal (45°) and, particularly, the transverse (90°) orientations introduced more interruptions and transitions between layers, increasing the contact resistance and reducing the overall conductivity compared to the longitudinal one (0°). They could be proposed in applications where a balance between moderate conductivity and mechanical reinforcement across layers is desired. Additionally, the longitudinally printed samples displayed a relatively stable resistance across a range of temperatures, while the diagonal and transverse configurations showed an increase in resistance at elevated temperatures, indicating a positive temperature coefficient (PTC) effect. The increased thermal stability could potentially extend the operational lifespan of 4D-printed devices made from 6 wt% GNP/PVDF composite in high-temperature applications. The ability to modify these properties through the adjustment of processing parameters, such as printing direction and layer thickness, highlights the potential for optimizing material performance. The higher heating efficiency at a low power consumption demonstrate the effectiveness of the thin-film design in optimizing thermal performance and energy efficiency in comparison to the thicker 3D-printed film. Future studies will focus on understanding the mechanisms underlying the thermal, electrical, and self-heating behaviors in detail, possibly leading to the development of materials specifically designed to harness the benefits of graphene reinforcement and 3D printing while maintaining optimal performance under varying operational conditions. Overall, the insights gained from this study contribute to the ongoing exploration of PVDF/GNP nanocomposites as suitable candidates for next-generation 3D-printable electronic devices for thermo-resistive applications.

## Figures and Tables

**Table 1:** Thermal analyses—DSC and TGA/DTG results: *T_m_*, melting temperature; Δ*H_m_*/ω, normalized heat enthalpy; *χ*_C_, percent of crystallinity; *T_onset_*, onset temperature of degradation; and *T_peak_*, peak of the first derivative.

Sample	DSC—First Heating Run, After 3D Printing				DSC Cooling	DSC—Second Heating Run			TGA/DTG	
	T_m1_, °C	T_m_, °C	ΔH_m_/ω, Jg^−1^	*χ*_C1_, %	T_c_, °C	T_m_, C	=H_m_/ω, Jg^−1^	*χ*_C2_, %	T_onset_, °C	T_DTGpeak_, °C
PVDF	167.5	171.2	57.4	54.8	139.7	169.9	60.6	57.9	449.1	466.3
6GNP/PVDF	163.5	169.5	48.7	46.5	141.4	168.7	53.2	50.8	453.8	474.5
GNP	-	-	-	-	-	-	-	-	605.7	694.3

**Table 2:** The resistive heating characteristics and electrical resistance (R), resistivity (ρ), and conductivity (σ) of the 6 wt% GNP/PVDF samples of a thickness of 2 mm, when 3D printed in three directions, with raster angles of 0°, 45°, and 90°.

Printing Direction	Voltage (V)	T_max_ (°C)	I_msax_ (A)	P (W)	H (J)	H_r_ (°C/s)	H_eff_ (%)	ΔT (°C)	R (Ω)	ρ (Ω·m)	σ (S·m^−1^)
Longitudinal, 3DP 0°	2	64.6	0.225	0.45	225	0.45	61	39.6	8.9	0.0089	112
Diagonal, 3DP 45°	2	53.7	0.180	0.36	180	0.22	53	28.7	11.1	0.0111	90.1
Transverse, 3DP 90°	2	44.9	0.137	0.27	135	0.15	44	19.9	14.6	0.0146	68.5

**Table 3:** Summary of Joule heating characteristics for 4 printed layers (0.8 mm thick) and 10 printed layers (2 mm thick) of 3DP 45° samples, varying the applied voltage.

Printed Layers	Voltage (V)	T_max_ (°C)	I_max_ (A)	P (W)	H (J)	H_r_ (°C·s^−1^)	H_eff_ (%)	ΔT, °C
10	2	53.0	0.192	0.38	114	0.20	53	28.0
10	3	78.0	0.289	0.87	261	0.43	68	53.0
10	4	109.0	0.378	1.51	453	0.65	77	84.0
4	2	32.0	0.040	0.08	24	0.15	22	7.0
4	3	42.9	0.066	0.20	60	0.21	42	17.9
4	4	54.4	0.088	0.35	105	0.25	54	29.4
4	5	69.6	0.109	0.55	165	0.43	64	44.6
4	7	92.0	0.146	1.02	306	0.65	73	67.0

## Data Availability

The original contributions presented in this study are included in this article. Further inquiries can be directed to the corresponding author.
